# Intertidal estimates of sea urchin abundance reveal congruence in spatial structure for a guild of consumers

**DOI:** 10.1002/ece3.7958

**Published:** 2021-08-09

**Authors:** Kevin C. K. Ma, Suzanne Redelinghuys, Molline N. C. Gusha, Siphelele B. Dyantyi, Christopher D. McQuaid, Francesca Porri

**Affiliations:** ^1^ Department of Zoology and Entomology Rhodes University Grahamstown South Africa; ^2^ South African Institute for Aquatic Biodiversity Grahamstown South Africa

**Keywords:** co‐occurrence, echinoderm, echinoids, rocky shores, South Africa, spatial heterogeneity, spatial scales, spectral analysis

## Abstract

We hypothesized congruence in the spatial structure of abundance data sampled across multiple scales for an ecological guild of consumers that exploit similar nutritional and habitat resources. We tested this hypothesis on the spatial organization of abundance of an herbivorous guild of sea urchins. We also examined whether the amount of local along‐shore rocky habitat can explain the observed spatial patterns of abundance. Standardized estimates of abundance of four intertidal sea urchins—*Diadema* cf. *savignyi*, *Echinometra mathaei*, *Parechinus angulosus*, and *Stomopneustes variolaris—*were determined by six observers at 105 sites across 2,850 km of coast of South Africa. For each species and observer, wavelet analysis was used on abundance estimates, after controlling for potential biases, to examine their spatial structure. The relationship between local sea urchin abundance and the amount of upstream and downstream rocky habitat, as defined by the prevailing ocean current, was also investigated. All species exhibited robust structure at scales of 75–220 km, despite variability among observers. Less robust structure in the abundances of three species was detected at larger scales of 430–898 km. Abundance estimates of sympatric populations of two species (*D*. cf. *savignyi* and *E. mathaei*) were positively correlated with the amount of rocky habitat upstream of the site, suggesting that upstream populations act as larval sources across a wide range of scales. No relationship between abundance and habitat size was found for *P. angulosus* or *S*. *variolaris*. Within the range of scales examined, we found robust congruence in spatial structure in abundance at the lower, but not the larger, range of scales for all four species. The relationship between abundance and upstream habitat availability in two species suggests that larval supply from upstream populations was probably the mechanism linking habitat size and abundance.

## Graphical abstract

We hypothesized and demonstrated congruence in the spatial structure of abundance data sampled across multiple scales for an ecological guild of consumers that exploit similar nutritional and habitat resources. We tested this hypothesis on the spatial organization of abundance of an herbivorous guild of intertidal sea urchins across 2,850 km of coast of South Africa. We also examined whether the amount of local along‐shore rocky habitat can explain the observed spatial patterns of abundance, which was the case for two of four focal species.

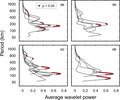

## 1 INTRODUCTION

Describing and disentangling the effects of the abiotic (e.g., environmental filtering) and biotic (dispersal, species interactions) factors that drive species distribution and abundance is a key focus of ecological and biogeographical research (Boulangeat et al., 2012; D'Amen et al., 2018; Gotelli & Ulrich, 2012). As an outcome of these interactive effects, three general distributional patterns can emerge for any pair of species in an assemblage: random, aggregated (co‐occurrence), and segregated (co‐exclusion or mis‐matched distribution; Charnov et al., 1976; Gotelli & Ulrich, 2012; Pitta et al., 2012; Veech, 2014). In many assemblages, co‐occurrence is not commonly encountered, even among pairs of congeneric species, where limited or absence of competition should theoretically allow for strong positive spatial association (Pitta et al., 2012; Sfenthourakis et al., 2006). Instead, co‐occurrence is most frequently found in positive non‐trophic interactions (e.g., commensalism, facilitation) and less so in trophic (e.g., predation) and negative non‐trophic interactions (e.g., competition), suggesting that spatial patterns in occurrence can be used to infer general species interactions (Freilich et al., 2018).

Consumers belonging to the same ecological guild typically exploit and compete for similar food and habitat resources, exhibiting negative non‐trophic interactions. In contrast, other forms of intraguild interactions (e.g., mutualism) could result in strong spatial association, that is, co‐occurrence patterns across the landscape or seascape (Crowley & Cox, 2011). Conversely, intraguild predation (a form of trophic interaction) is expected to lead to spatial segregation between predator and prey (McPeek, 2014). Unlike intraguild predation, intraguild mutualism (e.g., interactions within guilds of herbivores) has received much less attention in the literature. Because competitive exclusion is expected among herbivores competing for the same resources, spatial co‐occurrence among such species suggests some form of mutualism or facilitative interaction (Assaneo et al., 2013; Crowley & Cox, 2011).

Sea urchins (Echinodermata: Echinoidea) are slow‐moving grazers in intertidal and subtidal marine systems. Although they can feed opportunistically on marine invertebrates and biofilm, they primarily feed on macroalgae (Freeman, 2006; Loiderios & Gracía, 2006; Russell et al., 2018; Saucede et al., 2006) and are keystone species in the structuring of kelp forest communities (Estes & Palmisano, 1974; Scheibling et al., 1999). The collapse of algal‐dominated communities through over‐grazing has given rise to so‐called urchin barrens as an alternative stable state in many places (Filbee‐Dexter & Scheibling, 2014), often with a sharp “grazing front” between kelp beds and urchin barrens (Gagnon et al., 2004). Overgrazing is frequently associated with dramatic increases in urchin abundances through reduced urchin predation, reflecting an inverse relationship between predator and prey abundances (Brown‐Saracino et al., 2007; McClanahan, 1998).

Sea urchins generally undergo long‐distance dispersal during their planktonic larval life stage, which lasts from weeks to several months (Cram, 1971; Dautov, 2020; Huggett et al., 2005; Strathmann, 1978). The influence of environmental filtering on sea urchin abundance has been documented along various environmental stress gradients including wave exposure and pH (Baggini et al., 2015; Lamb et al., 2020). These environmental parameters reduce the efficiency or ability of sea urchins to search for and consume food and, in turn, lower their growth rates, fecundity, and/or survival, hence contributing to the determination of their local and regional abundances and the limits of their ranges. In addition, environmental thermal regimes can affect ontogenic development of sea urchins, their dispersal abilities, and, in turn, recruitment into the adult habitat (O'Connor et al., 2007; Rahman et al., 2007), further influencing the spatial structures of populations.

Because they are grazers, the abundances of sea urchin are likely to reflect local availability of food (e.g., algae) and other environmental parameters influencing food availability (e.g., temperature, wave exposure). We therefore expected spatial structure in abundances at local scales of 10s to 100s of km, rather than large (≥100s km) biogeographic effects. We tested this by describing the present distributional ranges of four intertidal sea urchins in South Africa, evaluating the presence of spatial structure in their abundances, and identifying the dominant spatial scales of their distributions. Given that they are likely to compete for the same nutritional and habitat resources, we assessed the generality of this expectation by testing for congruent spatial patterns among the species. Next, we tested the prediction that the abundances of members of the same guild (in this case predominantly herbivores) would exhibit inverse relationships due to competition (Crowley & Cox, 2011). To do this, we examined the relationships (positive, inverse, or no relationships) between the abundances of each pair of urchins where they were sympatric. Lastly, we tested the general assumption that upstream larval supply and the direction and range of dispersal as determined by the prevailing coastal current. Thus, downstream urchin abundance would be positively correlated with available upstream rocky shore habitat, which acts a proxy for larval sources.

## 2 MATERIALS AND METHODS

### 2.1 Field surveys

**FIGURE 1:**
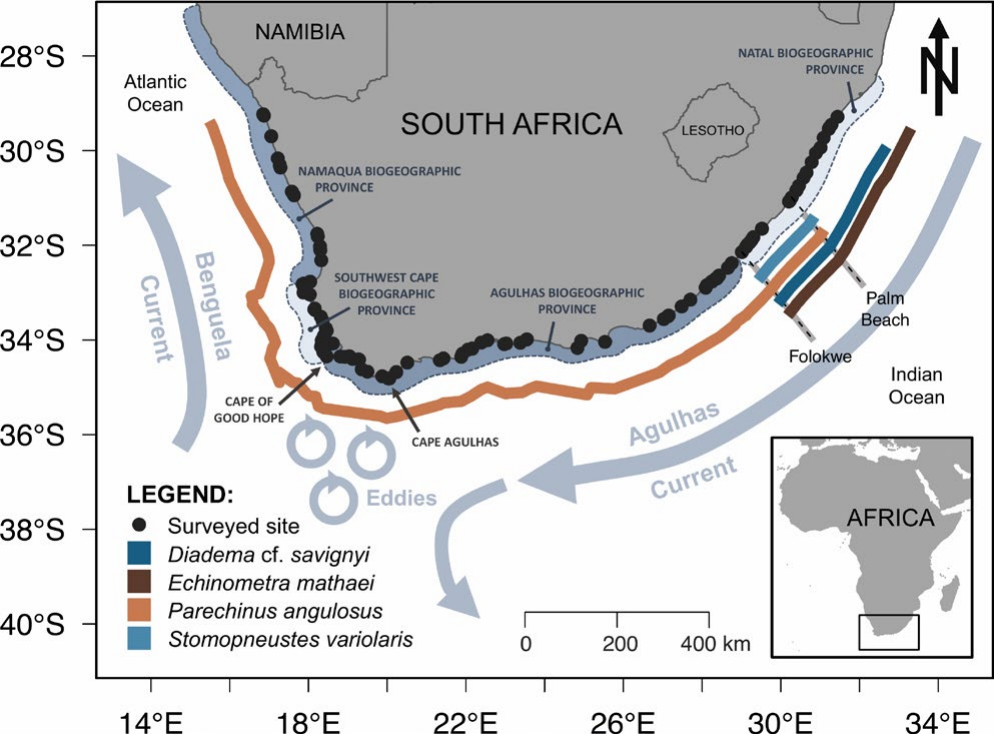
Locations of surveyed sites (*n* = 105 sites after removing sites with potential observer biases; solid black points) and the South African distribution of four intertidal sea urchin species in 2019–2020: *Diadema* cf. *savignyi* (dark blue line), *Echinometra mathaei* (dark brown line), *Parechinus angulosus* (light brown line), and *Stomopneustes variolaris* (light blue line)

From September 2019 to March 2020, a total 164 visits were made to 116 rocky shore sites (i.e., some sites were visited more than once; Figure 1) across 2,850 km of the South African coast from Alexanderbaai in the northwest (28.65°S; 16.48°E) to Zinkwazi Beach in the east (29.29°S; 31.44°E) in order to survey the distribution and abundance of four species of intertidal sea urchins: *Diadema* cf. *savignyi* (Audouin, 1809), *Echinometra mathaei* (Blainville, 1825), *Parechinus angulosus* (Leske, 1778), and *Stomopneustes variolaris* (Lamarck, 1816). A fifth species, *Tripneustes gratilla* (Linnaeus, 1758), was observed (*n* = 22 after 219 observer‐minutes of effort) from only one site and was not included in the study. For sites that were visited more than once, either different observer(s) surveyed the same site, but at different times, or a different area of the rocky shore was surveyed by the same observer(s). In particular, we used a handheld GPS unit to demarcate surveyed areas to ensure that we did not re‐survey the same area. We adopted a form of catch per unit effort (CPUE) mode of estimating abundances by standardizing the number of adult individuals of each species with a test diameter of 30 mm or larger to the time spent searching to give the number of individuals per observer‐minute.

The number of observers varied between one and four per site (a total of six unique observers trained as marine biologists) and total search time varied among sites (range: 8 min to 4.28 hr; mean: 0.83 hr). At each site, observers searched different areas of the rocky shore in a haphazard fashion with minimal spatial overlap to reduce the chance of double‐counting. With respect to time spent on the shore, one observer tended to focus, though not exclusively, on species detection at the midshore level (Observer #1), another observer at the low shore level and the lower fringe (Observer #2), and the remaining four observers over a wide vertical range from the low shore to the high shore levels (Observers #3 to #6). When there was a single observer on a shore, sea urchin abundance was surveyed over the entire vertical range of the intertidal zone. All observers have a similar level of taxonomic experience in detecting intertidal sea urchins (Fitzpatrick et al., 2009) and access to resources for species identification (Branch et al., 2010; Filander & Griffiths, 2017).

### 2.2 Removing potential biases

We accounted for three potential sources of sampling or observer bias that could affect detection: short sampling duration on some occasions, high water levels (tides), and inter‐observer differences. Other possible sources of bias, such as site topography (e.g., flat vs. steeply sloping habitats) and weather conditions (e.g., level of cloud cover), were beyond the scope of this study.

#### 2.2.1 Sampling duration

Visits that involved a sampling duration (a proxy for search effort) of less than 15 min were eliminated from the analyses because sea urchins were consistently not detected (false negative results) from these sites (a total of 11 visits were excluded on this criterion). Negative results for visits longer or equal to 15 min, which are more likely to be true negative results, were retained in the dataset.

#### 2.2.2 Tidal height

Spring tidal range throughout the study area is ca. 2 m, and sampling for sea urchins was done during spring low tides, however the minimum height of the tide during sampling differed among sites, ranging from 0.09 to 1.43 m (mean ± *SD*: 0.48 ± 0.22 m). For each site, tidal height at the start and end of sampling was determined based on predicted hourly tide charts (Kampfer, 2017, 2018). Because high tidal height during sampling might bias species detection by making this difficult, we removed all visits from our analyses that involved sampling when tidal height was greater than 0.83 m (a total of eight visits).

Thus, to reduce the possibility of false negatives due to (a) short sampling duration (six visits), (b) high tides (three visits), and (c) both short sampling duration and high tides (five visits), a total of 11 sites were removed from our dataset, leaving 105 unique sites that were used in our analyses (Figure 1).

#### 2.2.3 Observer bias

To account for differences among observers in their ability to detect sea urchins, we evaluated whether abundance data independently determined by any two observers from different parts (i.e., areas of shore) of the same three or more shores (i.e., sites) were positively correlated. The nonparametric Spearman rank‐order correlation coefficient was used because the distribution of abundance values was right‐skewed. Due to high inter‐observer variability in counts among shared sites, individual spatial analyses were done for the data from each of the six observers (*n* = 25–101 sites, depending on the observer).

### 2.3 Data analyses

Where species were sympatric, correlations between the mean abundances of pairs of the four sea urchin species (a total of six pairwise combinations) were evaluated with the Spearman rank‐order correlation coefficient because the data were heavily skewed to the right. Spatial autocorrelation of log_10_ (*x* + 1) transformed abundance across its South African range was determined using global spatial statistics (Moran's *I*) for each of the four species. Distances between adjacent sites were transformed from GPS coordinates in Cartesian space to their along‐shore distances (in kilometers) along a straight line. To account for the geometry of the coastline, this transformation ensures that distances between adjacent sites do not intersect with any landmasses. Resulting variograms were examined visually to assess the spatial variability in sea urchin abundance as a function of distance. Because variograms decompose at large lag intervals, 80% of the maximum lag interval (ca. 165 km for *S*. *variolaris*, ca. 370 km for *D*. cf. *savignyi* and *E. mathaei*, and ca. 2,000 km for *P*. *angulosus*) was used as our largest lag interval in the analyses (Pérez‐Castañeda & Defeo, 2004).

Spatial structure in abundance (i.e., detectable repeating patterns) across different scales was examined using a wavelet analysis (Fortin and Dale, 2005; Ma et al., 2021). Wavelet analyses have typically been used to detect patterns in time‐series datasets (e.g., Walter et al., 2020), but were applied here to a spatial signal, that is, patterns in sea urchin abundance along their linear range in South Africa. For each species, the wavelet power spectrum was generated with the Morlet wavelet (100 simulations). The resulting spectrum was summarized by plotting the average wavelet power for each period representing spatial scales ranging from 50 to 1,600 km.

The Agulhas Current and the Benguela Current are the main ocean currents influencing hydrodynamics for most coastal regions of South Africa, with prevailing offshore circulation patterns from northeast to southwest for sites influenced by the Agulhas Current and from south to north for sites influenced by the Benguela Current (Figure 1). Within the distributional range of each species, the relationship between amount of local along‐shore rocky habitat (habitat size) and mean abundance of the four studied sea urchin species was evaluated with the Spearman rank‐order correlation coefficient. The presence or absence of along‐shore rocky habitat was annotated across approximately 3,190 km of coastline of South Africa at 1‐km intervals by visual examination of the most current satellite imagery available from Google Earth Version 9.3.115.1 (https://www.google.com/earth/). The amount (along‐shore distance) of local rocky habitat upstream and downstream, relative to the dominant current (Agulhas or Benguela) on that stretch of coast, was estimated for each of our 105 sites using spatial windows ranging from 5 to 140 km at 5‐km intervals for each direction (upstream and downstream). This resulted in a total of 28 spatial windows of amount of habitat for each direction to be correlated with mean abundance at each site. To account for the use of multiple comparisons, we used the Benjamini–Hochberg adjusted significance level, a method which nominally assumes that individual tests are independent but (for our analyses) can be general enough for tests that are dependent of each other (Benjamini & Yekutieli, 2001). Given that *P*. *angulosus* was distributed across more than one biogeographic province, the analysis of the relationship between abundance and local amount of rocky habitat was also partitioned by biogeographic province (namely, Namaqua, Southwest Cape, Agulhas, and Natal; Figure 1) to separate the potential effects of different prevailing oceanic currents on larval dispersal distance. All figures and analyses were done in the R programming environment (R Core Team, 2020).

## 3 RESULTS

### 3.1 Distribution and abundance

**FIGURE 2:**
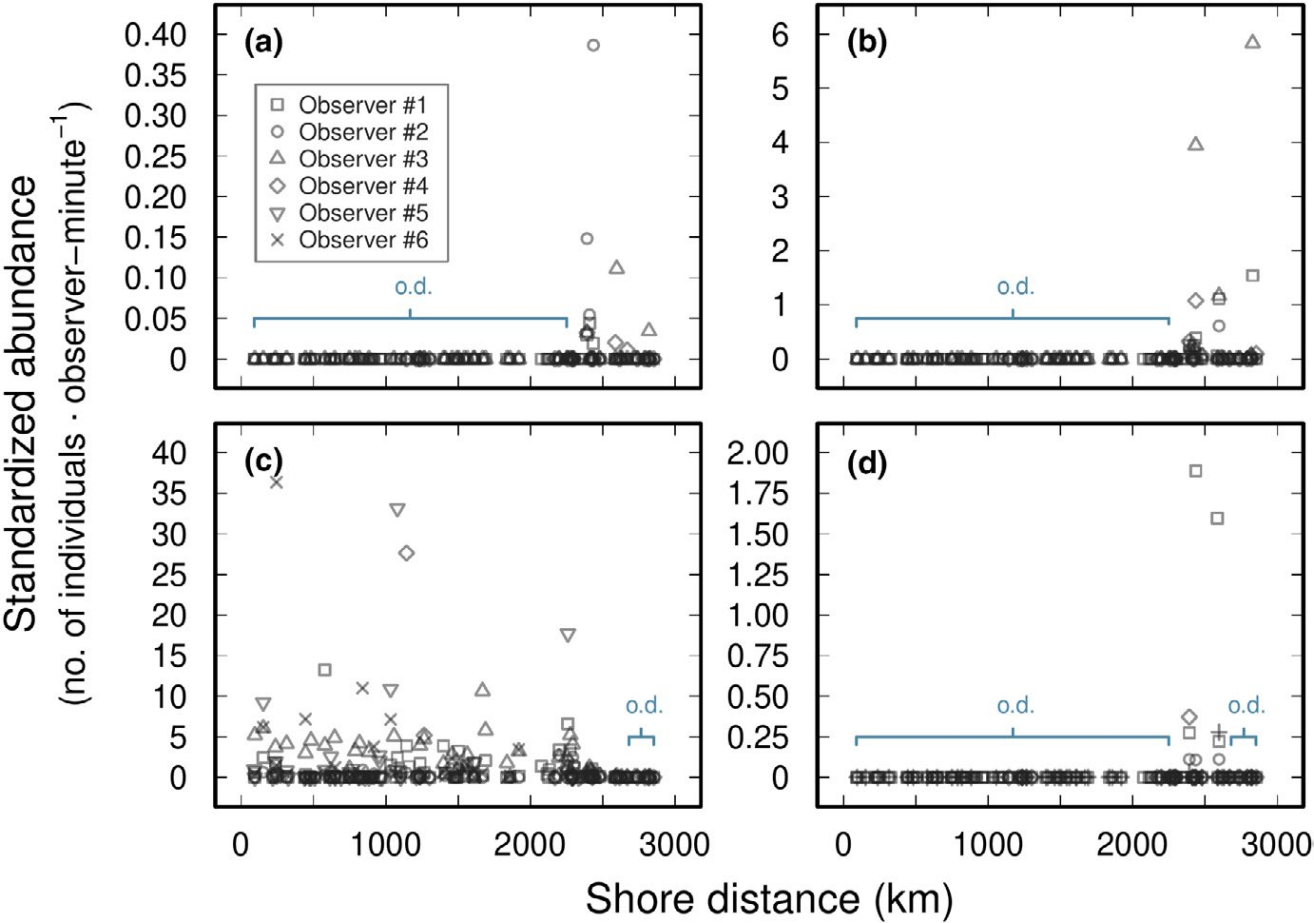
Separate abundance estimates of intertidal sea urchins across coastal South Africa for each observer: (a) *Diadema* cf. *savignyi* (*n* = 4 observers), (b) *Echinometra mathaei* (*n* = 4), (c) *Parechinus angulosus* (*n* = 6), and (d) *Stomopneustes variolaris* (*n* = 4); shore distance of 0 km starts at the mouth of the Orange River at the border of South Africa and Namibia; o.d. = outside of distribution, where the species was not detected by all observers; note that *y*‐axis ranges are different for each panel

Of the four species inspected, *Parechinus angulosus* had the widest distribution, spanning all four biogeographic provinces of the South African coast (Namaqua, Southwest Cape, Agulhas, and Natal) defined by Lombard et al. (2004; Figure 1). The three other species—*Diadema* cf. *savignyi*, *Echinometra mathaei*, and *Stomopneustes variolaris*—all had smaller ranges restricted to the Natal province (Figure 1). During sampling at low tide, *D*. cf. *savignyi*, *E*. *mathaei*, *P*. *angulosus*, and *S*. *variolaris* tended to be found in rock pools or, sometimes, exposed to air at the subtidal fringe. Although it was also found in rock pools, *S*. *variolaris* was frequently found higher up the intertidal zone in crevices that are exposed to air during low tide. Abundance (standardized by search time) of each species varied geographically along the coast (Figure 2). Within their respective ranges, *P*. *angulosus* was the most abundant (mean of 1.35 individuals observer‐minute^−1^; *n* = 92 sites), followed by *S*. *variolaris* (0.28 individuals observer‐minute^−1^; *n* = 10 sites), *E*. *mathaei* (0.28 individuals observer‐minute^−1^; *n* = 23 sites), and *D*. cf. *savignyi* (0.01 individuals observer‐minute^−1^; *n* = 21 sites).

All four sea urchin species were sympatric over 205 km of shore along the southeast coast from Folokwe to Palm Beach; Figure 1). In the region where *D*. cf. *savignyi* and *E*. *mathaei* were both present, abundances of the two species within sites were positively correlated (Spearman's rho = 0.47, *p* < .001). Within the region where all four species co‐occurred, pairwise mean abundances of (a) *D*. cf. *savignyi* and *E*. *mathaei* and (b) *E. mathaei* and *S*. *variolaris* were significantly positively correlated (Table 1), while the relationship between *D*. cf. *savignyi* and *S*. *variolaris* abundance estimates was close to statistical significance (Spearman's rho = 0.60, *p* = .052). In contrast, mean *P*. *angulosus* abundance within this sympatric range did not correlate with mean abundances of any other intertidal sea urchin species (Table 1).

**TABLE 1:** Spearman's correlation coefficients (rho) matrix of mean abundance of four intertidal sea urchin species (*Diadema* cf. *savignyi*, *Echinometra mathaei*, *Parechinus angulosus*, and *Stomopneustes variolaris*) observed in the sympatric range from Folokwe to Palm Beach, South Africa (*n* = 12 sites); n.s. = not statistically significant; *p < .05; **p < .01; ***p < .001

Species	*D*. cf. *savignyi*	*E. mathaei*	*P. angulosus*	*S. variolaris*
*D*. cf. *savignyi*	–	0.67*	n.s.	n.s.
*E. mathaei*		–	n.s.	0.63*
*P. angulosus*			–	n.s.
*S. variolaris*				–

### 3.2 Spatial structure in abundance

Because there was an overall lack of correlation in abundance estimates by pairs of observers, spatial autocorrelations in abundance (global Moran's *I*) and wavelet analyses for each sea urchin species were performed on the data of each observer separately. Wavelet analyses detected spatial structure in the form of repeating patterns in abundance for all four species by least two observers per species (Table 2).

**TABLE 2:** Summary of the dominant spatial scale detected by the average (global) wavelet power spectra of abundance estimates of four intertidal sea urchins across coastal South Africa: *Diadema* cf. *savignyi*, *Echinometra mathaei*, *Parechinus angulosus*, and *Stomopneustes variolaris*; n.a. = not applicable because there were no significant patterns detected

Observer	Focal intertidal zone	Estimated spatial extent (km)	No. of sites sampled (*n*)	Dominant spatial scale (km)			
				*D*. cf. *savignyi*	*E. mathaei*	*P. angulosus*	*S. variolaris*
Observer #1	Mid shore	2,763.5	101	120^a^	n.a.	754	75^c^
Observer #2	Low shore/lower fringe	2,759.0	61	212	176	n.a.	120
Observer #3	Entire vertical range	2,734.5	48	168	n.a.	220^b^	n.a.
Observer #4	Entire vertical range	1,710.5	25	164	198	n.a.	n.a.
Observer #5	Entire vertical range	2,260.5	45	–	–	121	–
Observer #6	Entire vertical range	1,825.0	42	–	–	n.a.	–

^a^ Secondary dominant spatial scale ranged from 430 to 898 km.

^b^ Secondary dominant spatial scale at 577 km.

^c^ Secondary dominant spatial scale at 609 km.

With respect to the lack of correlation in abundance estimates by pairs of observers, only four pairs of observers out of a total of 24 pairwise combinations (10 combinations for *P*. *angulosus*, six for *D*. cf. *savignyi*, six for *E*. *mathaei*, and two for *S*. *variolaris*), correlated positively. Although it is reasonable to expect an absence of correlation between pairs of observers who surveyed different shore heights, the lack of correlation also included pairs of observers who surveyed a similar range of shore heights across different areas of the same site.

**FIGURE A1:**
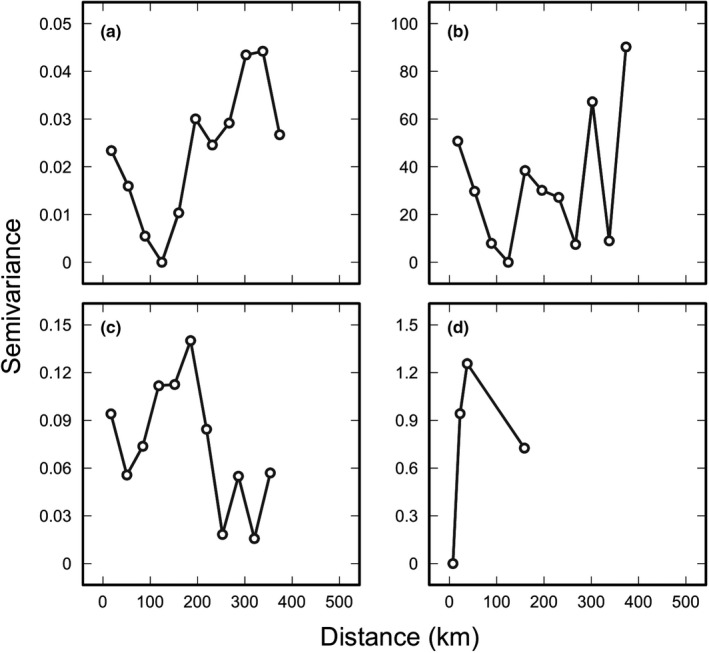
Observed variograms of log_10_ (*x* + 1) transformed abundance estimates of *Diadema* cf. *savignyi* across coastal South Africa based on data from (a) observer #1, (b) observer #2, (c) observer #3, and (d) observer #4; no data collected for this species from observers #5 and #6; note that *y*‐axis ranges are different for each panel

**FIGURE A2:**
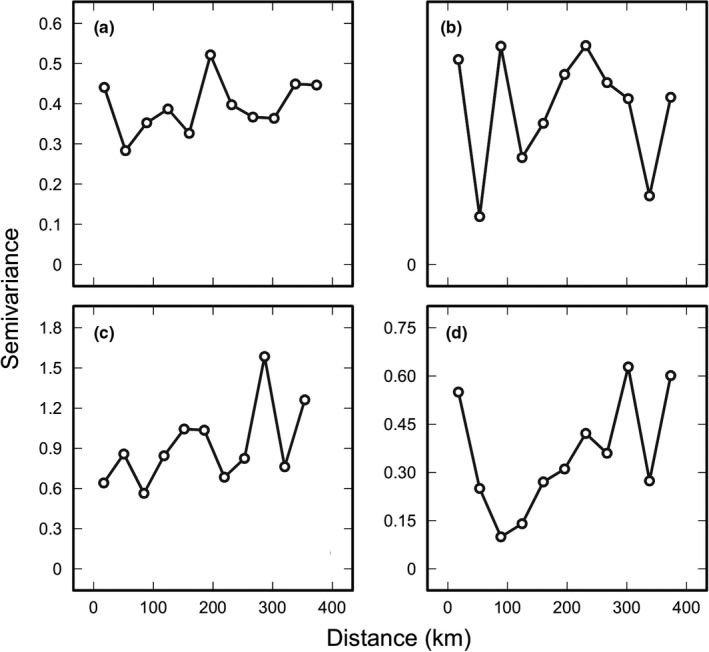
Observed variograms of log_10_ (*x* + 1) transformed abundance estimates of *Echinometra mathaei* across coastal South Africa based on data from (a) observer #1, (b) observer #2, (c) observer #3, and (d) observer #4; no data collected for this species from observers #5 and #6; note that *y*‐axis ranges are different for each panel

**FIGURE A3:**
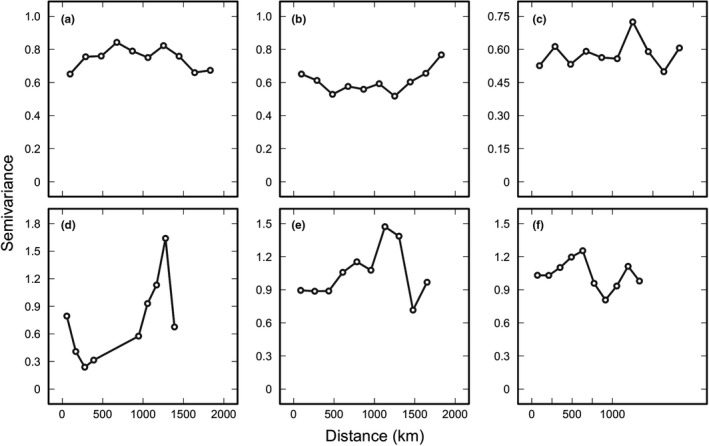
Observed variograms of log_10_ (*x* + 1) transformed abundance estimates of *Parechinus angulosus* across coastal South Africa based on data from (a) observer #1, (b) observer #2, (c) observer #3, (d) observer #4, (e) observer #5, and (f) observer #6; note that *y*‐axis ranges are different for each panel

**FIGURE A4:**
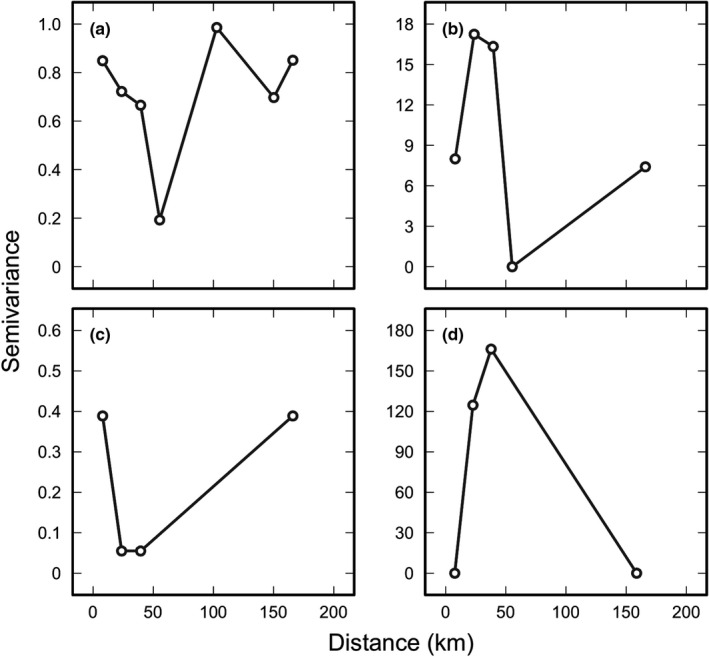
Observed variograms of log_10_ (*x* + 1) transformed abundance estimates of *Stomopneustes variolaris* across coastal South Africa based on data from (a) observer #1, (b) observer #2, (c) observer #3, and (d) observer #4; no data collected for this species from observers #5 and #6; note that *y*‐axis ranges are different for each panel

None of the four sea urchin species exhibited clear, consistent patterns of spatial autocorrelation of abundance within its range (Table A1 and Figures A1, A2, A3, A4 in the Appendix A). Specifically, no autocorrelations were found in abundance estimates of *D*. cf. *savignyi*, *E*. *mathaei*, or *S*. *variolaris*, indicating random spatial patterns (Table A1 in the Appendix A). Of the six individual observer‐based datasets of *P*. *angulosus* abundance, autocorrelation (positive *z*‐score of 0.23) was detected only in the dataset made by observer #3 reflecting the fact that abundance estimates were spatially clustered (Table A1 in the Appendix A).

**FIGURE 3:**
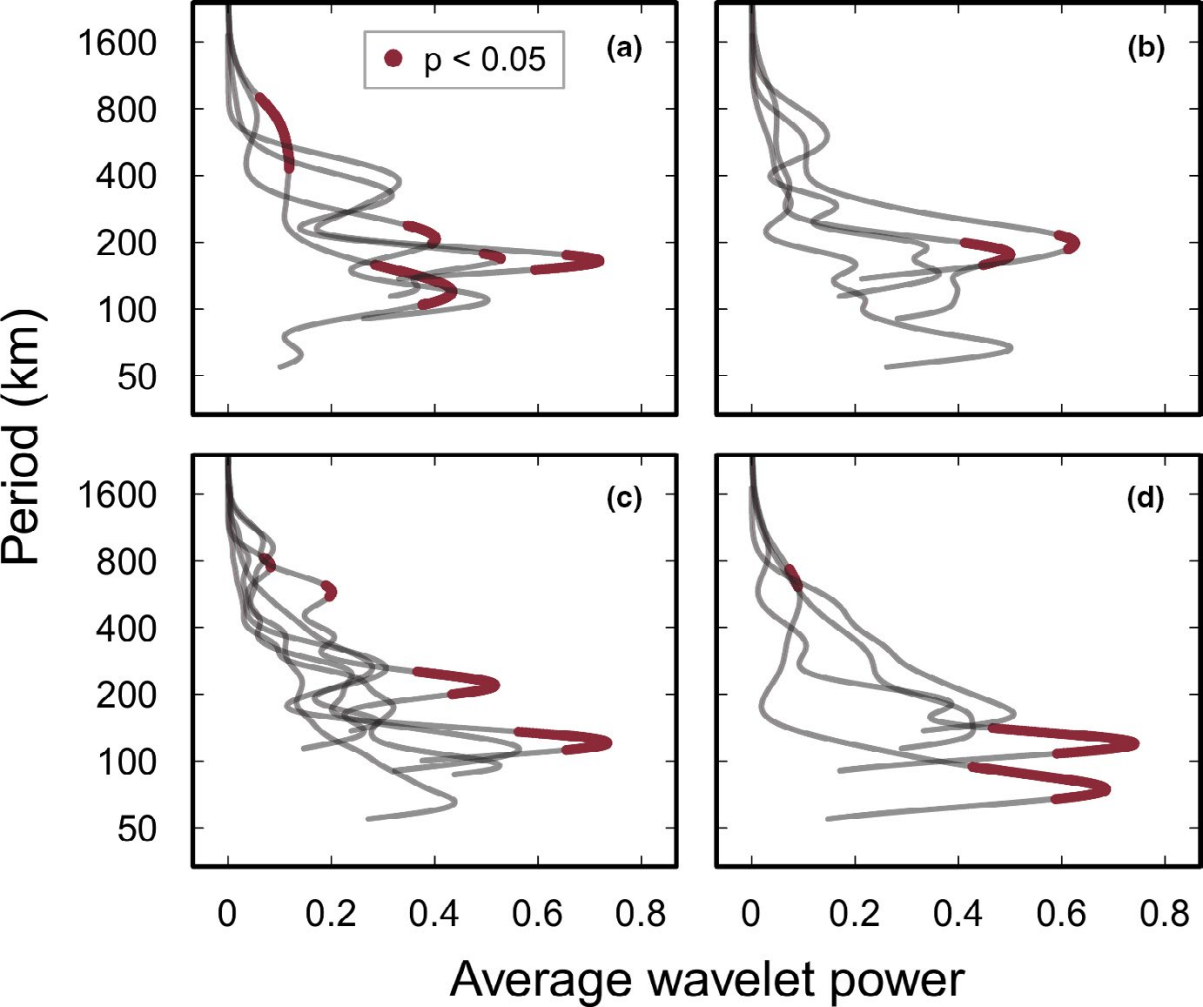
Separate average (global) wavelet power spectra of mean abundance of intertidal sea urchins across coastal South Africa for each observer: (a) *Diadema* cf. *savignyi* (*n* = 4 observers), (b) *Echinometra mathaei* (*n* = 4), (c) *Parechinus angulosus* (*n* = 6), and (d) *Stomopneustes variolaris* (*n* = 4); NB: *y*‐axes are plotted on a logarithm scale (base 2)

Observer‐specific wavelet analyses revealed some differences among observers in the dominant spatial scales of sea urchin abundance (Table 2 and Figure 3). Eleven of the 18 analyses (i.e., one species was detected by all six observers and three other species by only four observers) revealed significant repeating patterns in abundance at dominant scales ranging from 75 to 754 km and at secondary dominant scales from 430 to 898 km, but no detectable patterns for the remaining seven analyses (Table 2). Overall, significant spatial patterns on scales ranging from 75 to 220 km were relatively robust, with average wavelet power of >0.3 across all four species (Figure 3). However, significant spatial patterns on scales ranging from 430 to 898 km, detected in three species (namely, *D*. cf. *savignyi, P*. *angulosus*, and *S*. *variolaris*), were less robust (average wavelet power of <0.2; Figure 3). Wavelet analyses for each of the four observers who detected *D*. cf. *savignyi* revealed significant spatial patterns in abundance, but only 50% of the datasets for *E*. *mathaei* (two out of four observers) revealed patterns. Similarly, significant patterns were found in three out of six datasets for *P*. *angulosus* and two out of four datasets for *S*. *variolaris*. Specifically, robust spatial structure in urchin abundance was detected at scales between 120 and 212 km in *D*. cf. *savignyi*, between 176 and 198 km in *E*. *mathaei*, between 121 and 220 km in *P. angulosus*, and between 75 and 120 km in *S*. *variolaris* (Table 2 and Figure 3). Detectable, yet less robust spatial structure in abundance was found at larger scales of 430–898 km for *D*. cf. *savignyi*, of 577 and 753 km for *P*. *angulosus*, and of 609 km for *S*. *variolaris* (Table 2 and Figure 3).

### 3.3 Relationship with local rocky habitat

**FIGURE 4:**
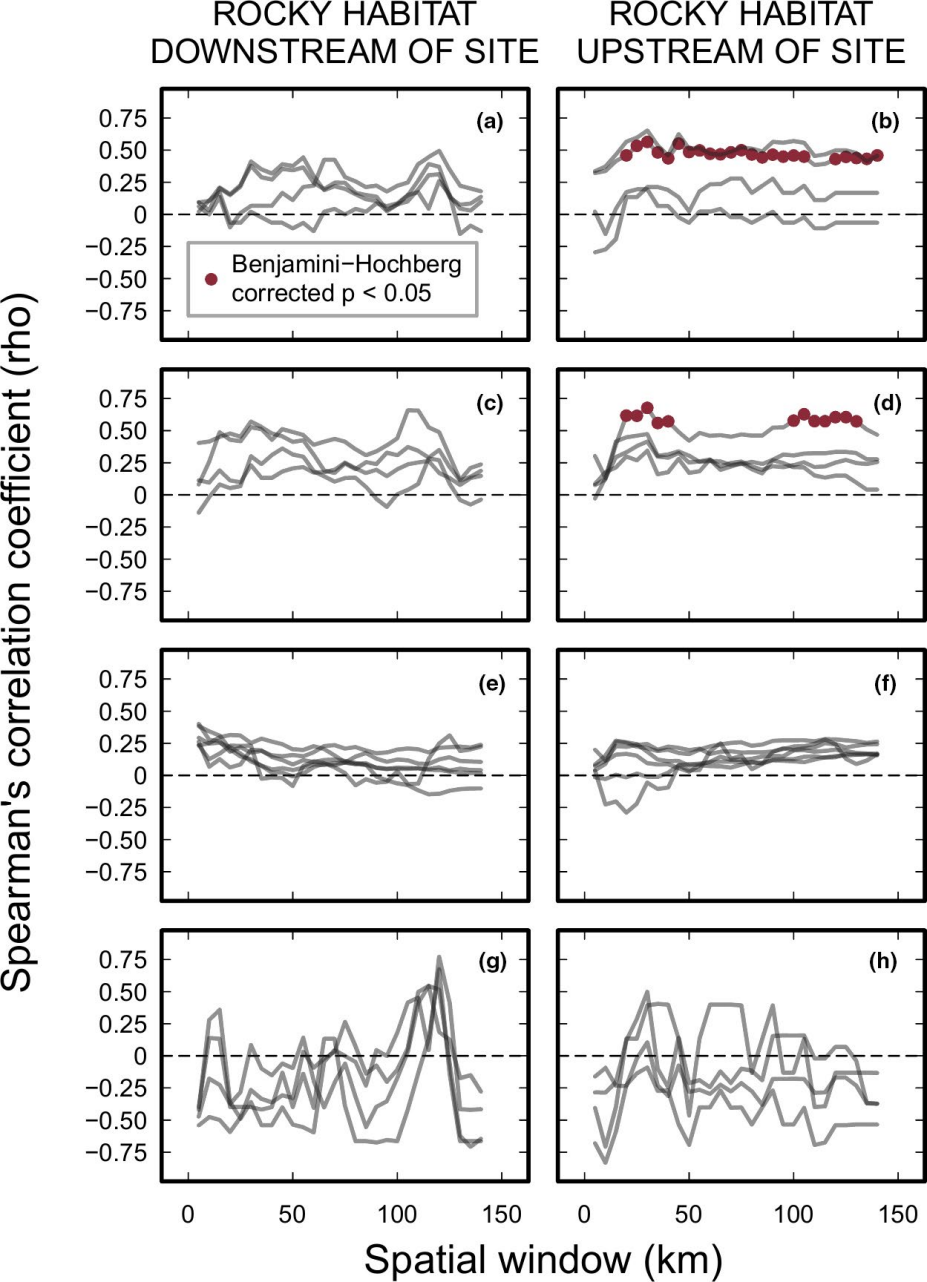
Relationship between the amount of local along‐shore rocky habitat, separated by rocky habitat downstream and upstream (with respect to the prevailing oceanic current) of the sampling site, and abundance estimates of four intertidal sea urchin species within the distributional range of each species: (a, b) *Diadema* cf. *savignyi* (*n* = 4 observers), (c, d) *Echinometra mathaei* (*n* = 4), (e, f) *Parechinus angulosus* (*n* = 6), and (g, h) *Stomopneustes variolaris* (*n* = 4); spatial window used to calculate the amount of rocky habitat ranged from 5 to 140 km at 5‐km intervals for each direction (downstream and upstream); horizontal dashed lines indicate where Spearman's rho = 0

**FIGURE A5:**
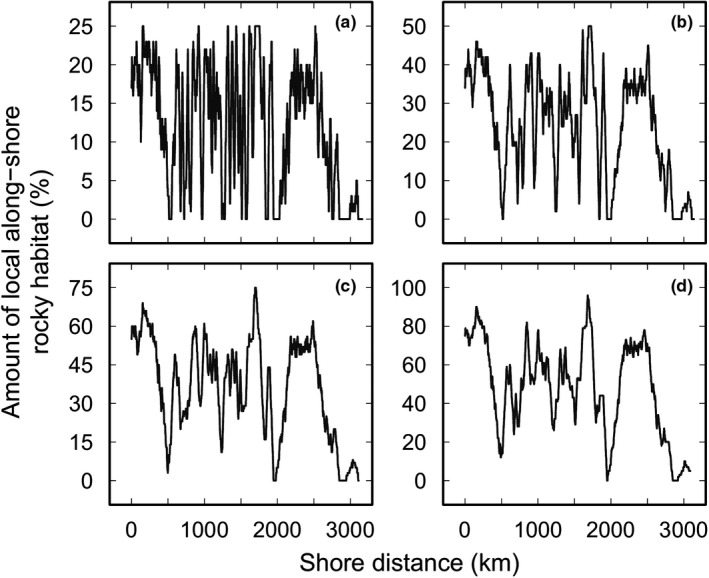
Distribution of rocky habitat along the coast of South Africa; moving average of the amount of habitat per spatial window of (a) 25 km, (b) 50 km, (c) 75 km, and (d) 100 km; shore distance of 0 km starts at the mouth of the Orange River at the border of South Africa and Namibia

The amount of along‐shore rocky habitat (habitat size) differed around the coastline of South Africa, and the running average of the amount of habitat is smoothed with increasing size of the spatial window (Figure A5 in the Appendix A). For *D*. cf. *savignyi*, *E*. *mathaei*, and *P*. *angulosus*, the relationship between abundance estimates and the amount of local rocky habitat tended to be positively correlated (Figure 4). However, there were no clear relationships (i.e., spasmodic fluctuations between positive and negative correlations) for *S*. *variolaris*, regardless of which observer's dataset was used (Figure 4).

Abundances of both *D*. cf. savignyi and *E*. *mathaei* were significantly, positively correlated with the amount of upstream rocky habitat (Figure 4). In the case of *D*. cf. *savignyi*, urchin abundance correlated with the amount of rocky habitat extending 20–140 km upstream (with respected to the dominant current direction) of the sampling site based on the data collected by only one observer (i.e., observer #1; Table A2 in the Appendix A). Abundance of *E*. *mathaei* and local rocky habitat extending 20–130 km upstream correlated positively, again, based on data of one observer (i.e., observer #2; Table A2 in the Appendix A). No relationship, however, was found between abundance and the amount of local rocky habitat for *P*. *angulosus* or *S*. *variolaris* (Table A2 in the Appendix A).

## 4 DISCUSSION

The ranges of four targeted sea urchins along the South African coastline differed among species and matched with known contemporary biogeographic boundaries (Emanuel et al., 1992; Spalding et al., 2007; Teske et al., 2011): *Parechinus angulosus* being restricted to cold and warm temperate waters, *Diadema* cf. *savignyi* and *Echinometra mathaei* to subtropical waters, and *Stomopneustes variolaris* to the region transitioning from warm temperate to subtropical waters (about 205 km of shore; Stephenson, 1944; Day, 1969; Marshall et al., 1991). Although *P*. *angulosus* has the widest distribution in South Africa, the ranges of *D*. *savignyi* and *E*. *mathaei* extend further northeast in the Indian Ocean, beyond the borders of South Africa, while *P*. *angulosus* also occurs further to the northwest in the Atlantic Ocean (Day, 1969). The extensive distributions of *D*. cf. *savignyi*, *E*. *mathaei*, and *P*. *angulosus* are consistent with the tendency of sea urchins to have larger ranges—at scales of 1,000s of km—than other marine invertebrates (Emlet, 1995). Further, sea urchin ranges can extend about 7,000 km (mean distance) for species with planktotrophic larvae, which includes all four species in the present study (Cram, 1971; Dautov, 2020; Drummond, 1991; Emlet, 1995; Rahman et al., 2007). The limited range of *S*. *variolaris* in South Africa, however, may reflect a disjunct distribution when viewed from a broader scale since the species, like *D*. cf. *savignyi* and *E*. *mathaei*, is known from subtropical localities in the Indian Ocean (Drummond, 1991).

Our findings reveal modest shifts in the South African range edges when our distributional descriptions were compared to earlier data: a cold‐edge (along‐shore) expansion of ca. 45 km southwest for *D*. cf. *savignyi* and *S*. *variolaris*, a cold‐edge contraction of ca. 100 km northeast for *E*. *mathaei* unless the species shifted exclusively to the subtidal over this distance, and a warm‐edge contraction of ca. 220 km southwest for *P*. *angulosus* (Day, 1969; Farquhar, 1994; Marshall et al., 1991). In fact, the range shifts observed in the present study represent changes of only 0°15′ toward the South Pole in the expansion of *D*. cf. *savignyi* and *S*. *variolaris*, 0°35′ toward the Equator in the contraction of *E*. *mathaei*, and 1°27′ toward the South Pole in the contraction of *P*. *angulosus*. Despite the small distances in poleward range shifts, these patterns are likely part of a global trend strongly linked to ocean warming in many marine invertebrates and macroalgae (Lenoir et al., 2020; Nicastro et al., 2013; Pinsky et al., 2013). Additional southern African records of *P*. *angulosus* (a seemingly temperate‐adapted species), however, have been documented further northeast in subtropical waters (undated records; Filander & Griffiths, 2017), suggesting either a significant gap in distribution between the temperate (western) and subtropical (eastern) populations or that the subtropical population is restricted to the subtidal zone and, unlike the western population, not typically found in the intertidal zone.

This study provides a detailed account of along‐shore variability in relative urchin abundances by using a CPUE‐like approach, that is, standardizing abundance by duration of search time. Although CPUE measures of urchin abundance (i.e., weight of harvest per duration time effort) tend to be positively related to other measures of densities (e.g., numbers per m^2^), CPUE is generally considered a poor predictor of density (i.e., low *R* ^2^ values relating CPUE and urchin density; Schroeter et al., 2009). Yet, the use of CPUE as a relative measure of urchin abundance can be efficiently repeated by many human observers and upscaled to many sites and regions around the world to understand macro‐ecological patterns at multiple scales. A majority of our pairwise abundance estimates were positively related between a majority of our observers; however, statistically significant correlations were only found between a few pairs of observers. This lack of correlation in CPUE‐like estimates among observers suggests that other unaccounted sources of bias may contribute to inter‐observer variation in highly stratified habitats such as rocky shores. For instance, the distribution of sea urchins is likely to vary across the vertical (and horizontal) range of the intertidal, which may explain the lack of correlation among observers. In particular, surveys from two observers (e.g., Observers #1 and #2) were biased toward sampling lower on the shore.

Our findings show that all four species exhibited clear spatial structure (i.e., repeating patterns of abundance) at local scales between 75 and 220 km within their respective South African ranges. This suggests congruence in spatial structure in abundance estimates at local scales for these four species, which supports our hypothesis for generality in this spatial structure for a guild of herbivores that compete for similar food and habitat resources (i.e., food and habitat availability are expected to drive their distribution and abundance). Additionally, our data indicate weaker spatial structure in *D*. cf. *savignyi*, *P*. *angulosus*, and *S*. *variolaris* abundance at larger scales between 430 and 900 km, indicating that different ecological processes likely structure urchin abundance at local and at large scales.

Elsewhere around the world, spatial patchiness in urchin densities across multiple scales has frequently been observed in many species (Brown‐Saracino et al., 2007; Casal et al., 2020; Hasan, 2019; Morgan et al., 2000; Sánchez‐Jérez et al., 2001; Vanderklift & Kendrick, 2004; Wing, 2009). The tendency for individual sea urchins to aggregate at varying densities and patch sizes, coupled with habitat heterogeneity, contributes greatly to this spatial variability in abundance (Dumas et al., 2007; Freeman, 2003; Ouréns et al., 2015). Moreover, Morgan et al. (2000) did not find any relationship between urchin recruitment and adult densities in populations of *Strongylocentrotus franciscanus* in California, which suggests that larval dispersal (including cross‐shore and along‐shore transport), instead, determines urchin distribution and abundance. Conversely, other studies have found a positive relationship when suitable microhabitat (e.g., holdfast, spines of conspecific adults) for recruitment was available, as this is likely to increase postsettlement survival (Hunt & Scheibling, 1997; Ouréns et al., 2014; Palleiro‐Nayar et al., 2011; Tegner & Dayton, 1977). From longer‐term studies, sporadic and infrequent (pulsed) recruitment events have been observed in some urchin populations and contribute to substantial spatio‐temporal variability in the size‐frequency structure of urchin populations (Pearse & Hines, 1987). Coastal sea urchin fisheries tend to occur at small‐scales (i.e., smaller than the scales examined in the present study), and spatial heterogeneity (i.e., spatial autocorrelation) at scales of about 180–350 m has been found in an Italian fishery (Addis et al., 2009), though not at comparable scales of 10s to 100s of m in an American fishery, despite substantial spatial variability in densities (Grabowski et al., 2005).

Site‐level co‐occurrence of sea urchin species (from two to four species) on the east coast of South Africa is likely to be due to either similar habitat requirements or facilitative interactions within this guild of herbivores (Crowley & Cox, 2011). Several models of interspecific interactions can explain facilitation among sea urchin species. For instance, the consumption of food resources by one herbivore may increase food production, benefiting other herbivores (Crowley & Cox, 2011). Another possible model may be the simultaneous exploitation of resources (e.g., food, space), which re‐engineers the habitat and consequently reduces stressors exerted on the species or improves the environmental conditions they experience (Crowley & Cox, 2011). In particular, species in the genus *Echinometra* can bio‐erode coral reef habitats and reduce its complexity, which facilitates co‐existence with other, large herbivorous species (Brown‐Saracino et al., 2007; McClanahan & Muthiga, 2013). Both cases are examples of resource‐mediated direct mutualism. Accordingly, an outcome of these forms of mutualism would be positive correlation between consumer species. Yet, such strong spatial association due to mutualism and/or environmental suitability is likely to explain correlations between only two of the six species pairs that we examined. Although no known predators of sea urchins have been identified from the sympatric region on the east coast, predation of *P*. *angulosus* by the rock lobster, *Jasus lalandii* (H. Milne Edwards, 1837), on the west coast is notable in the literature (see Van Zyl et al. (2003) and references therein). In tropical systems, predation by coral reef fish of the competitively dominant sea urchin species can also maintain co‐existence of multiple sea urchin species, though this trophic interaction is increasingly disrupted by human fishing activities (Humphries et al., 2020; McClanahan, 1988). Also, antagonistic interactions, such as biting and pushing behavior, and direct competition for resources have been documented within and among sea urchin species (McClanahan & Muthiga, 2013; Shulman, 1990; Williams, 1981), and can be partially avoided through spatial niche partitioning. In particular, this spatial partitioning can result in species segregation by bathymetric depth (Tuya et al., 2007), by intertidal height on the rocky shore (Farquhar, 1994; Marshall et al., 1991), or by microhabitat type (e.g., crevices) on coral reefs (McClanahan, 1988).

Ocean circulation, which regulates temperature at the scale of biogeographic provinces (Smit et al., 2013), influences gene flow and population structure of *P*. *angulosus* in South Africa. Molecular analysis by Muller et al. (2012) revealed that the west coast population is relatively isolated, likely due to the effects of eddies generated by the Agulhas Current that are associated with strong phylogeographic breaks between Cape Agulhas and the Cape of Good Hope (Teske et al., 2011; Figure 1). In addition to inferences made from genetic connectivity of populations, the effect of ocean circulation on larval urchin dispersal has been inferred from latitudinal recruitment patterns on the west coast of the United States (Ebert & Russell, 1988; Morgan et al., 2000). In this study, we detected a relationship between local (downstream) abundances of *D*. cf. *savignyi* and *E*. *mathaei* and available (upstream) rocky shore habitat, which may serve as a proxy for larval sources. This assumes that rocky shore habitat is populated with reproductive individuals and that the amount of habitat is roughly proportional to population size. This suggests that there is a spatial relationship between larval source and the sampled population. In particular, our analysis suggests that larval sources for *D*. cf. *savignyi* and *E*. *mathaei* extended over a broad range of along‐shore distances from nearby (i.e., narrow spatial window of ca. 20 km upstream of site) to relatively far (i.e., larger spatial window of ca. 130–140 km upstream of site). Our findings, moreover, conspicuously feature a lack of relationship with available habitat for populations of *P*. *angulosus* and *S*. *variolaris*, and an absence of correspondence among observers where there were positive relationships between abundance estimates and upstream habitat size. The lack of such relationships suggests that *P*. *angulosus* and *S*. *variolaris* abundances may be decoupled from the effects of the prevailing oceanic current or, additionally, may be influenced by counter currents. The absence of correspondence among observers indicates that any significant positive relationships between urchin abundance and habitat availability represent a somewhat coarse ecological link between larval supply from an upstream source population and downstream abundance. Of course, taxon‐dependent timing (e.g., Carson et al., 2010) and duration of spawning, larval predation, pelagic larval duration, postsettlement processes, and prevailing and transient hydrodynamics will also affect larval supply and recruitment patterns, further shaping the realized distribution of adult populations. Without more complete information on the drivers and patterns of these early processes, it is difficult to draw further conclusions on how they help explain urchin distribution in South Africa.

In summary, the intertidal distribution of sea urchins in South Africa suggests minor range expansions and contractions in the last 25 years. It is, however, difficult to distinguish between along‐shore (horizontal) range contractions, where the species disappeared, and downslope (vertical) range shifts, where the species may withdraw from the intertidal, but persist in the subtidal. Observations of sea urchins in their sympatric intertidal range provide insights into the co‐existence of herbivores. For instance, a combination of mutualism among herbivores and spatial niche partitioning likely contributes to their co‐existence on the east coast of South Africa. This sympatric region also comprises genetically endemic populations of other species (e.g., the prawn *Kraussillichirus kraussi* (Stebbing, 1900)), which suggests that it is not just a region of faunal overlap, but there may be a yet unknown ecological process (e.g., environmental filtering) that could select particular genetic strains within a species, allowing it to establish and persist (Golla et al., 2020; but see Jooste et al., 2018). The detection of congruent spatial structure in abundance estimates at local scales (site‐level) for all four sea urchin species suggests that the same ecological processes at corresponding local scales are likely to regulate their population sizes and shape their distributions on rocky shores in South Africa. For this herbivorous guild, robust congruence in spatial structure at the local scale could, for instance, reflect the local availability of food resources (e.g., drift algae), and/or processes involved in larval dispersal (from production to transport to settlement). Spatial structure in abundance was less robustly detected at larger scales for three of the four species, which may reflect ecological process(es) (e.g., larval dilution by oceanic currents) different from those responsible for structure in abundance at local scales. A distinct relationship between the availability of upstream rocky habitat and downstream population size in *D*. cf. *savignyi* and *E*. *mathaei* suggests that coarse inferences about larval dispersal patterns could be made for some sea urchin species with planktotrophic larvae. Although all four species can also be found subtidally (down to 30 m in the case of *P. angulosus*; Branch et al., 2010), making inferences about larval supply more problematic, our data suggest that larval supply as influenced by upstream habitat size is a substantive supply‐side factor influencing population size.

## CONFLICT OF INTEREST

None declared.

## AUTHOR CONTRIBUTIONS

**Kevin C. K. Ma:** Conceptualization (lead); data curation (lead); formal analysis (lead); investigation (lead); methodology (lead); visualization (lead); writing‐original draft (lead); writing‐review & editing (lead). **Suzanne Redelinghuys:** Data curation (supporting); project administration (equal); writing‐review & editing (supporting). **Molline N. C. Gusha:** Data curation (supporting); project administration (equal); writing‐review & editing (supporting). **Siphelele B. Dyantyi:** Data curation (supporting); project administration (equal); writing‐review & editing (supporting). **Christopher D. McQuaid:** Supervision (equal); writing‐review & editing (equal). **Francesca Porri:** Supervision (equal); writing‐review & editing (equal).

## Data Availability

Data generated from this study are deposited online in Dryad and available at https://doi.org/10.5061/dryad.mkkwh7110.

## APPENDIX A

**TABLE A1:** Summary of the global Moran's *I* statistics on log_10_ (*x* + 1) transformed abundance estimates of four intertidal sea urchins within their respective range across coastal South Africa: *Diadema* cf. *savignyi*, *Echinometra mathaei*, *Parechinus angulosus*, and *Stomopneustes variolaris*; n.s. = not statistically significant; **p* < .05; ***p* < .01; ****p* < .001

Observer	*p* value (Moran's *I*)			
	*D*. cf. *savignyi*	*E. mathaei*	*P. angulosus*	*S. variolaris*
Observer #1	n.s.	n.s.	n.s.	n.s.
Observer #2	n.s.	n.s.	n.s.	n.s.
Observer #3	n.s.	n.s.	<0.001***	n.s.
Observer #4	n.s.	n.s.	n.s.	n.s.
Observer #5	–	–	n.s.	–
Observer #6	–	–	n.s.	–

**TABLE A2:** Distances upstream and downstream (with respect to the prevailing oceanic current) of the sampling site where significant positive relationships (Spearman rank‐order correlation coefficient) between the amount of local along‐shore rocky habitat and abundance estimates of four intertidal sea urchin species within the distributional range of each species: *Diadema* cf. *savignyi*, *Echinometra mathaei*, *Parechinus angulosus*, and *Stomopneustes variolaris*; n.s. = not statistically significant; observer #1 (*n* = 101 sites), observer #2 (*n* = 61), observer #3 (*n* = 48), observer #4 (*n* = 25), observer #5 (*n* = 45), observer #6 (*n* = 42)

Observer	Distance from the sampling site (km)			
	*D*. cf. *savignyi*	*E. mathaei*	*P. angulosus*	*S. variolaris*
Observer #1	20–105, 120–140 km upstream	n.s.	n.s.	n.s.
Observer #2	n.s.	20–40, 100–130 km upstream	n.s.	n.s.
Observer #3	n.s.	n.s.	n.s.	n.s.
Observer #4	n.s.	n.s.	n.s.	n.s.
Observer #5	–	–	n.s.	–
Observer #6	–	–	n.s.	–
